# Potential of primary kidney cells for somatic cell nuclear transfer mediated transgenesis in pig

**DOI:** 10.1186/1472-6750-12-84

**Published:** 2012-11-09

**Authors:** Anne Richter, Mayuko Kurome, Barbara Kessler, Valeri Zakhartchenko, Nikolai Klymiuk, Hiroshi Nagashima, Eckhard Wolf, Annegret Wuensch

**Affiliations:** 1 Chair for Molecular Animal Breeding and Biotechnology, and Laboratory for Functional Genome Analysis (LAFUGA), Gene Center, Ludwig-Maximilians-Universität München, Feodor-Lynen-Straße 25, Munich, 81377, Germany; 2 Meiji University International Institute for Bio-Resource Research, Kawasaki, Japan

**Keywords:** Pig, Primary kidney cells, Fibroblasts, Nuclear transfer, Genetic engineering

## Abstract

**Background:**

Somatic cell nuclear transfer (SCNT) is currently the most efficient and precise method to generate genetically tailored pig models for biomedical research. However, the efficiency of this approach is crucially dependent on the source of nuclear donor cells. In this study, we evaluate the potential of primary porcine kidney cells (PKCs) as cell source for SCNT, including their proliferation capacity, transfection efficiency, and capacity to support full term development of SCNT embryos after additive gene transfer or homologous recombination.

**Results:**

PKCs could be maintained in culture with stable karyotype for up to 71 passages, whereas porcine fetal fibroblasts (PFFs) and porcine ear fibroblasts (PEFs) could be hardly passaged more than 20 times. Compared with PFFs and PEFs, PKCs exhibited a higher proliferation rate and resulted in a 2-fold higher blastocyst rate after SCNT and *in vitro* cultivation. Among the four transfection methods tested with a GFP expression plasmid, best results were obtained with the Nucleofector^TM^ technology, resulting in transfection efficiencies of 70% to 89% with high fluorescence intensity, low cytotoxicity, good cell proliferation, and almost no morphological signs of cell stress. Usage of genetically modified PKCs in SCNT resulted in approximately 150 piglets carrying at least one of 18 different transgenes. Several of those pigs originated from PKCs that underwent homologous recombination and antibiotic selection before SCNT.

**Conclusion:**

The high proliferation capacity of PKCs facilitates the introduction of precise and complex genetic modifications *in vitro*. PKCs are thus a valuable cell source for the generation of porcine biomedical models by SCNT.

## Background

Over the last years the pig is getting more and more attractive as model organism for biomedical research due to similarities with humans in anatomy, size, physiology, metabolism and pathology (reviewed in [1]). Early sexual maturity and large litter sizes make pigs more suitable for research than other larger animals like cow, sheep or dog (reviewed in [2]). Furthermore, genetically engineered pigs are a promising source of cells, tissues and organs for xenotransplantation (reviewed in [3]).

Genetic modification of pigs can be achieved by a variety of approaches, including pronuclear microinjection of DNA, sperm-mediated gene transfer, retroviral transduction, and somatic cell nuclear transfer (SCNT) using transfected donor cells (reviewed in [4,5]). Due to the lack of germ line competent pluripotent stem cells in pigs, the latter approach is currently the only route for the generation of gene targeted pigs [6-8]. In addition, SCNT using pools of stable transfected cell clones was an efficient way for the production of transgenic founder pigs with appropriate expression patterns (reviewed in [1,9]) and facilitated the generation of the first pig models with inducible transgene expression [10].

Among the various parameters influencing the outcome of SCNT, such as oocyte quality, nuclear transfer protocol, embryo culture or recipient animal preparation, the type and quality of the nuclear donor cells are of vital importance (reviewed in [11]).

Many primary cell lines established after passaging a primary culture were used for SCNT in pigs, with differences regarding the cell type, source organ, age and gender of the donor animal. Among these cells, mesenchymal stem cells are more or less precisely characterized [12,13]. Fibroblast-like cells from fetuses, neonatal, juvenile or adult animals [14-16] or unspecified cells from different organs [17,18] are most widely used. These cells have been tested for their efficiency in SCNT experiments, but further characterization of the specific cell types, their morphology, proliferation, lifespan and stability of karyotype is mostly lacking. The latter parameters are particularly important for advanced genetic modification technologies such as gene targeting, where transfected cells need to undergo homologous recombination, clonal selection and proliferation up to a scale that provides a sufficient number of cells for recombination analysis, cryopreservation and subsequent SCNT experiments. The efficiency of genetic modification of primary cells also depends on the effective introduction of DNA vectors into the cells. Different methods for the transfection of primary mammalian cells have been described, including chemical (calcium phosphate precipitation [19], lipofection [20], nanofection [21]) and physical methods (electroporation [22], nucleofection [23], microinjection [24]) as well as viral transduction [25], but their applicability depends on the cell type into which the DNA has to be introduced.

Here, we characterize for the first time primary kidney cells (PKCs) isolated from kidney samples of juvenile pigs for their proliferation capacity, morphological appearance, stability of karyotype, and uptake of exogenous DNA after application of different transfection methods. Furthermore, we show their potential to support development of SCNT embryos *in vitro* and of viable genetically modified pigs after additive gene transfer and homologous recombination.

## Results

### Morphology and growth potential of PKCs compared to fibroblasts of different origin

**Figure 1:**
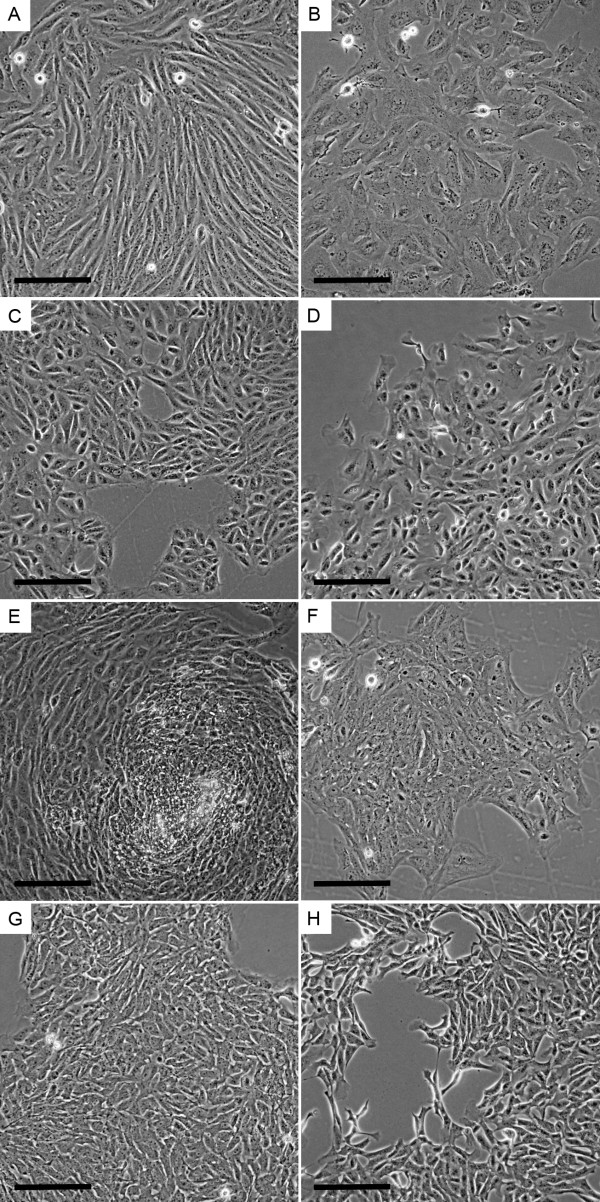
**Single cell clone colonies of PKCm at P3.** Single cell colonies were generated and analyzed after 7 days. The cells and formed colonies differed morphologically: fibroblast-like cells [**A**, **B**, **D**-**F**, **H**], epithelial- and endothelial-like cells [**C**, **G**], cell size (large [**B**], smaller [**A**, **C**-**H**]), colony compactness (cells very close [**A**, **E**, **G**], gaps between cells [**B**, **C**, **D**, **F**, **H**]) and colony shape (clearly defined [**A**, **C**, **E**, **G**], frayed colonies [**B**, **D**, **F**, **H**]). Scale bar = 100 μm.

Initial morphologic characterization of cells was performed 24 h after isolation by bright field microscopy. The cultures were usually 70–100% confluent and displayed a mixture of different cell morphologies. To characterize PKC diversity, the two kidney cell lines PKCm and PKC2109 were subcloned by generation of single cell clones at passage 3 and their morphology was evaluated 5 to 8 days later (Figure 1 and Additional file 1). The cell clones differed in morphology (fibroblast- or epithelial cell-like), cell size, colony formation (clearly defined or frayed), colony compactness (cell-to-cell distance), growth rate and lifespan.

**Figure 2:**
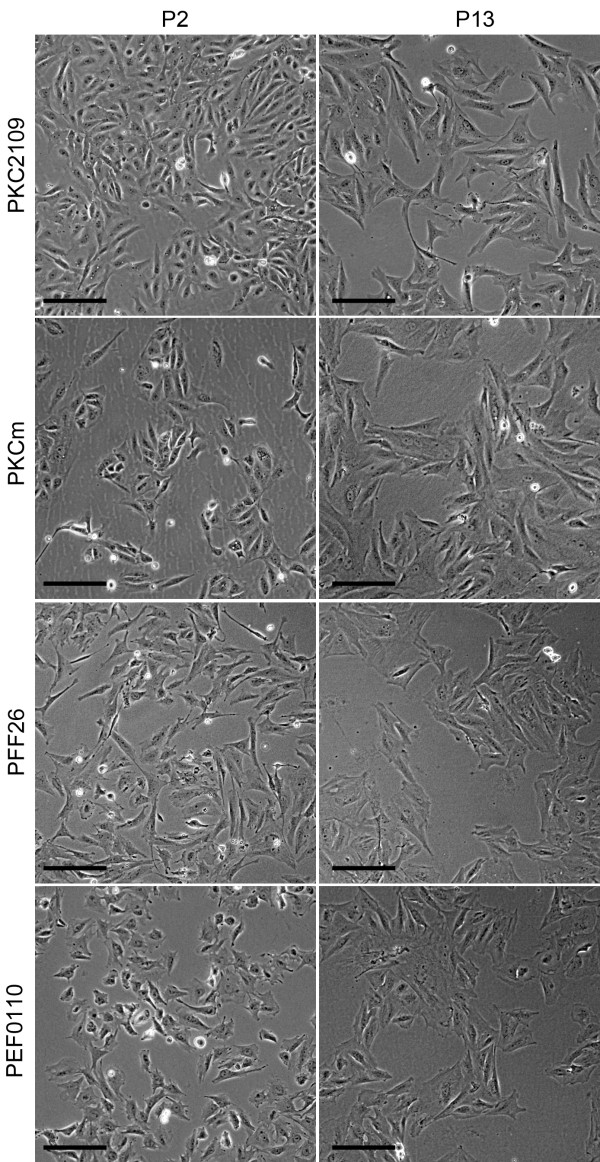
**Morphology of different primary pig cell lines after several passages.** Porcine kidney cells (PKCm and PKC2109), fetal fibroblasts (PFF26) and ear fibroblasts (PEF0110) showed all a more or less heterogeneous composition. In all primary cell lines the morphology changed from smaller and round-shaped in early passage (P2) to larger and long spindle-shaped cells in later passage (P13). Scale bar = 100 μm.

In parallel, we investigated porcine fetal fibroblasts (PFFs) and ear fibroblasts (PEFs) which also displayed morphological heterogeneity, though to a lesser extent than PKCs. In the primary cell lines PKCm, PKC2109, PFF26 and PEF0110, the heterogeneous appearance diminished with increasing passage numbers and the culture became dominated by cells with spindle shaped fibroblast-like morphology (Figure 2).

**Figure 3:**
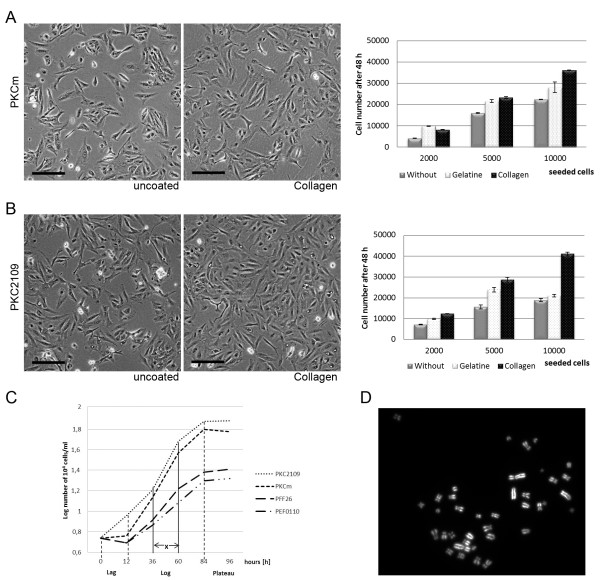
**Growth potential of porcine kidney cells compared to fetal and ear fibroblasts.** PKCm (**A**, left and middle panel) and PKC2109 (**B**, left and middle panel) grew rather in islands at P4 on non-coated plates compared to collagen-coated plates where they grew evenly spread. Scale bar = 100 μm. The proliferation assay showed best growth potential on collagen-coated plates with PKCm (**A**, right diagram) and PKC2109 (**B**, right diagram). (**C**) Growth curves of PKCm, PKC2109, PFF26 and PEF0110. Every 12–24 h cell numbers of various cell types were determined. The x-section (exponential growth phase) was used for calculation of population doubling time. PKC2109 and PKCm exhibited the shortest population doubling time compared to PFF26 and PEF0110. (**D**) Correct karyotype (2n=38) of PKC2109 at passage 71.

In a pilot experiment, PKCs seeded on non-coated cell culture plates did not get properly confluent and rather grew in islands. In contrast, on collagen-coated plates the cells grew evenly spread before reaching confluence (Figure 3A PKCm, B PKC2109). For systematic investigation of the growth behavior of PKCs on different coatings, the proliferation of the primary cell lines PKCm and PKC2109 was determined 48 h after seeding of different cell numbers (2,000, 5,000, or 10,000 cells per well) on gelatin-, collagen- or non-coated 96-well culture plates using an MTT based proliferation assay (Figure 3A and B right diagram). The collagen-coated plates notably promoted proliferation of the cells of both kidney cell lines, as well as PFF and PEF cells (data not shown). Therefore, all further culture experiments were performed in collagen-coated dishes.

Growth behavior and population doubling time of PKCm and PKC2109 was determined and compared to PFF26 and PEF0110 at passage 4–5. Growth curves were generated and population doubling time was calculated in the log phase (Figure 3C). After seeding, PKCm and in particular PKC2109 cells started earlier to proliferate and showed a steeper growth curve than PFF26 and PEF0110 cells. In the exponential growth phase, between 36 and 60 h after seeding (marked by an x in Figure 3C), the kidney cell lines exhibited the shortest population doubling time with 15.2 h (PKC2109) and 16.6 h (PKCm), followed by PFF26 (23.2 h) and PEF0110 (32.9 h).

In a long-term culture experiment, kidney cells were maintained for a high number of passages ‐ PKC2109 cells at least up to passage 71 and PKCm up to passage 50 ‐ showing only slight signs of senescence, whereas PFF and PEF could hardly be passaged more than 20 times. Karyotype analysis (Figure 3D) revealed 80% normal chromosome counts in PKCm at passage 3 and 74% in PKC2109 at passage 71, which was similar to the proportion of normal karyotypes in PFF26 (68%) and PEF0110 (75%) determined at passage 13. Due to the fact that it is difficult to prepare appropriate amounts of metaphases from cells with higher passages and cells with more than 12 passages are not used for SCNT in our lab we analyzed PFF26 and PEF0110 at passage 13.

### *In vitro* development of SCNT embryos derived from PKCs, PFFs, and PEFs

In 3 independent SCNT experiments PKCm, PFF26 and PEF0110 were used as donor cells (Table 1). Fusion rate and the proportion of development to blastocyst were significant higher with PKCm as donor cells compared to PEF0110. The cell numbers of blastocysts derived from PKCm, PFF26 and PEF0110 donor cells corresponded to 43.5±4.2, 56.1±9.5 and 23.5±7.5, respectively, and were not significantly different.

**Table 1:** ***In vitro *development competence of cloned embryos using PKCm, PEF0110 and PFF26**

**Donor cells**	**Fusion Rate (%)**	**Reconstructed embryos cultured**	**Reconstructed embryos developed to blastocyst stage (%)**				**Cell number of blastocysts [mean+/−SEM]**
			**hatched**	**not hatched**	**degen.**	**overall**	
PKCm	85/90	81	10	6	1	17 (21.0)^a^	43.5±4.2
	(94.4)^a^						
PEF0110	70/85	70	2	1	0	3 (4.3)^b^	23.5±7.5
	(82.4)^b^						
PFF26	74/86	77	5	2	0	7 (9.1)^a,b^	56.1±9.5
	(86.0)^a,b^						

Analysis of blastocysts on day 7. Values with different superscript were significantly different (P<0.05), SEM = standard deviation. degen.=degenerated.

### Genetic modification of primary PKCs

#### Chemical transfection methods

Lipofection of PKC2109 resulted in transfection efficiencies of 24 to 52% using 0.5 μg pmaxGFP^TM^ DNA, 0.5 to 1 μl Plus reagent and a lipid to DNA ratio of 2.5:1 or 3:1 (Table 2). Thereby, acceptable fluorescence intensities (score: ++) were observed, although serious proportions of detached cells indicated a high number of dead cells (rating: 1 to 2) and the attached cells were strongly spindle-shaped/partially enlarged and showed vacuoles/lipids (rating: 3 to 5). In the control approach (0 μg DNA, 2.5× lipid to DNA and 0.5 μl Plus reagent) cells had a good quality (rating: 1 to 2) without vacuoles and no detached cells (rating: 0; data not shown).

**Table 2:** Lipofection results PKC2109

**DNA amount [μg]**	**Plus reagent [μl]**	**Ratio lipid to DNA**	**Quality**	**Cells in suspension**	**Fluor. intensity**	**Transfection efficiency [%]**
**0.25**	0.25	1.0:1	1	0	+	**0.6**
		2.0:1	2	0	+	**0.5**
		2.5:1	2	0	+	**3.1**
		3.0:1	2	0	+	**3.8**
		4.0:1	4	0	++	**26**
**0.5**	0.5	1.0:1	2	0	+	**2.6**
		2.0:1	4	1	+	**26**
		2.5:1	3	1	++	**50**
		3.0:1	5	2	++	**52**
**0.5**	1.0	2.5:1	4	1	++	**24**
		3.0:1	4	2	++	**45**
**0.75**	0.75	1.0:1	4	0	++	**28**
		2.0:1	5	1	++	**37**
		2.5:1	5	1	++	**28**
		3.0:1	5	1	++	**21**

Scale for the assessment of cell quality: **1**- excellent to good; **2**- no vacuoles and lipids detectable, cells are bit stretched; **3**- partially vacuoles and lipids, stretched cells; **4**- many vacuoles and lipids, stressed cells, partially enlarged cells; **5**- a lot of large vacuoles and lipids, altered morphology and enlarged cells; Scale for the assessment of cells in suspension: **0**- none; **1**- low number of cells; **2**- a lot of cells; Fluor. = Fluorescence.

The highest nanofection efficiencies (20 to 25%) of PKC2109 were obtained using 1 μg of DNA and 1.2, 3.2 or 4 μl of Nanofectin (Table 3). In these approaches, cell quality (rating: 2) and the proportion of detached cells (rating: 1) were acceptable, but the fluorescence intensity was low when compared to lipofection (score: + to rarely ++). The control approaches (0 μg DNA, 3.2 μl Nanofectin and 1 μg DNA, 0 μl Nanofectin, respectively) resulted in excellent to good cell quality (rating: 1) and no cells in suspension (rating: 0, data not shown).

**Table 3:** Nanofection results PKC2109

**DNA amount [μg]**	**Nanofectin [μl]**	**Quality**	**Cells in suspension**	**Fluor. intensity**	**Transfection efficiency [%]**
0.5	1.2	1	0	+	**10**
	2.0	1	0	+	**16**
	3.2	1	1	+	**16**
	4.0	1	1	+	**15**
1.0	1.2	2	1	+	**25**
	2.0	2	1	+	**17**
	3.2	2	1	++	**21**
	4.0	3	1	+	**20**
1.5	2.0	3	1	+	**7**
	3.2	3	1	++	**11**

Scale for the assessment of cell quality: **1**- excellent to good; **2**- no vacuoles and lipids detectable, cells are bit stretched; **3**- partially vacuoles and lipids, stretched cells; Scale for the assessment of cells in suspension: **0**- none; **1**- low number of cells; **2**- a lot of cells; Fluor. = Fluorescence.

#### Physical transfection methods

To obtain optimal electroporation conditions for PKC2109, different DNA concentrations (1, 5, 10, 20 μg per 0.5×10^6^ and 1×10^6^ cells, respectively), resuspension buffers (electroporation buffer from Bio-Rad, DMEM, PBS) and voltages (100 V or 230 V) were tested (Table 4). The greatest transfection efficiencies (30 to 54%) were determined when using 0.5×10^6^ cells (5 or 10 μg DNA) and 1×10^6^ cells (10 or 20 μg DNA) at 230 V. Fluorescence intensity was very good, but a high proportion of cells did not attach after electroporation and indicated a high rate of cell loss during this process (rating: 2). Attached cells showed signs of stress including extreme spindle-shaped morphology and vacuoles (rating: 3 to 5). The control experiment (1×10^6^ cells, 0 μg DNA, electroporation buffer) resulted in stressed cells exhibiting large spindle-shaped morphology (rating: 4), but a lower number of cells did not attach (rating: 1; data not shown).

**Table 4:** Electroporation results PKC2109

**Cell number**	**DNA amount [μg]**	**Quality**	**Cells in suspension**	**Fluor. intensity**	**Transfection efficiency [%]**
0.5x10^6^	1	3	1	+	**28**
	5	3	2	+	**46**
	10	3	2	++	**47**
1.0x10^6^	1	4	2	+	**24**
	5	4	2	+	**22**
	10	4	2	++	**30**
	20	5	2	+++	**54**

Scale for the assessment of cell quality: **1**- excellent to good; **2**- cells are bit stretched/strongly fibroblast-like; **3**- stretched cells with vacuoles; **4**- very spindle-shaped morphology **5**- extreme spindle-shaped cells with vacuoles; Scale for the assessment of cells in suspension: **0**- none; **1**- low number of cells; **2**- a lot of cells; Fluor. = Fluorescence. Endotoxin-free purified DNA was used; Voltage: 230V.

**Figure 4:**
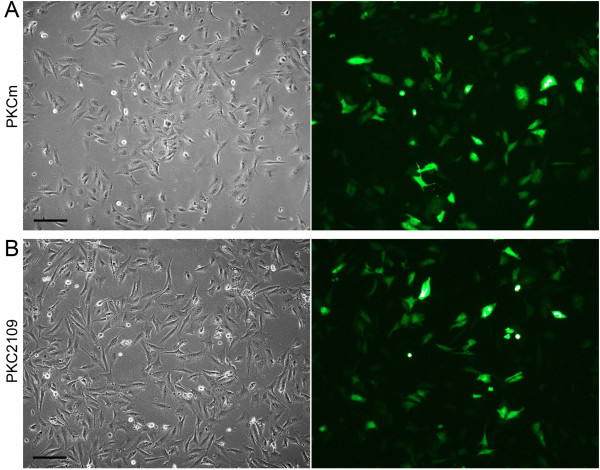
**Transiently nucleofected porcine kidney cells.** PKCm (**A**) and PKC2109 (**B**) 24 h after nucleofection with a GFP expressing plasmid using 0.5×10^6^ cells and 2 μg DNA at passage 4. Scale bar = 100 μm.

Optimal nucleofection parameters were ascertained in initial experiments by testing various programs offered by the supplier. Program U12 was the most efficient resulting in 63% transfection efficiency, good fluorescence intensity (score: ++) and cells of good quality (rating: 1) using 0.5×10^6^ cells of PKC2109, 2 μg DNA and 5 different nucleofection programs (Table 5). Therefore, U12 was used for further experiments using different cell numbers and quantity of DNA. Best transfection efficiency (89%), high fluorescence intensity (score ++), low number of cells in suspension (rating: 1) and good quality of cell morphology (rating: 2) was achieved using 0.5×10^6^ cells and 20 μg DNA. The control experiment (0.5×10^6^ cells of PKC2109, 0 μg DNA) resulted in best cell quality (rating: 1) and a low number of cells in suspension (rating: 1; data not shown). In PKCm, another primary kidney cell line, transfection efficiencies between 49% and 66% and high fluorescence intensity (score: ++ to +++) were determined using 0.5×10^6^ cells and 2 μg DNA. Representative pictures of nucleofected PKCs after using 2 μg DNA, 0.5×10^6^ cells and U12 are shown in Figure 4.

**Table 5:** Nucleofection results PKC2109, PKCm, PFF26 and PEF0110

**Cell Number**	**DNA amount [μg]**	**Program**	**Quality**	**Cells in suspension**	**Fluor. intensity**	**Transfection efficiency [%]**
**PKC2109**						
0.5x10^6^	2	A24	2	1	+	**28**
	2	U23	2	2	+	**60**
	2	U12	1	1	++	**63**
	2	T16	2	1	+	**54**
	2	V13	1	1	++	**59**
0.5x10^6^	1	U12	1	1	+	**36**
	5	U12	1	1	++	**70**
	10	U12	1	1	+++	**77**
	20	U12	2	1	++	**89**
1.0x10^6^	5	U12	1	1	++	**68**
	10	U12	1	1	+++	**88**
	20	U12	2	2	+++	**83**
**PKCm**						
0.5x10^6^	2	U12	1	1	++	**49**
	2	T16	1	1	++	**50**
	2	V13	2	1	+++	**66**
**PFF26**						
0.5x10^6^	2	U12	1	1	+	**53**
	2	T16	1	1	+	**50**
	2	V13	2	2	++	**77**
**PEF0110**						
0.5x10^6^	2	U12	1	1	+	**15**
	2	T16	1	1	+	**11**
	2	V13	2	2	+	**34**

Scale for the assessment of cell quality: **1**- excellent-good; **2**- cells are bit stretched/strongly fibroblast-like; **3**- stretched cells with vacuoles; **4**- cells with very spindle-shaped morphology **5**- extreme spindle-shaped with vacuoles; Scale for the assessment of cells in suspension: **0**- none; **1**- low number of cells; **2**- a lot of cells; Fluor. = Fluorescence.

After testing of 3 different nucleofection programs, U12 was the most efficient program for PFF26, due to transfection efficiency of 53%, excellent cell quality (rating: 1) and low number of cells in suspension (rating: 1).

Transfection of the primary cell line PEF0110 using 3 different nucleofection programs resulted in poor transfection efficiencies (11 to 34%) and low fluorescence intensity (score: +).

Among the different transfection methods used for transient transfection of PKC2109, nucleofection is the most suitable, since it is highly efficient and results in viable cells of good quality.

### Generation of transgenic animals using PKCs as donor cells

#### Additive gene transfer and re-cloning

**Figure 5:**
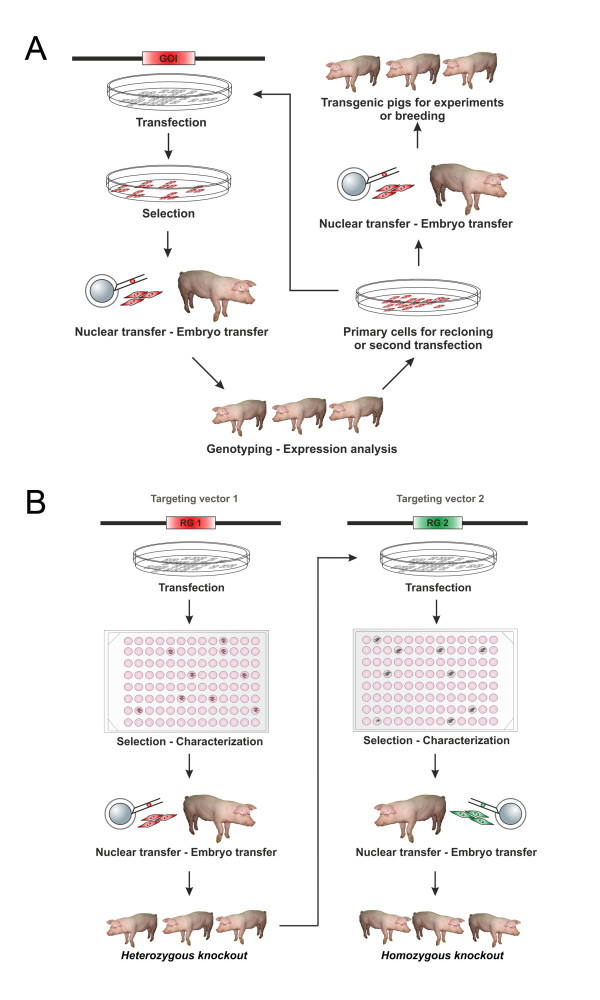
**Generation of transgenic pigs by additive gene transfer and gene targeting combined with SCNT.** (**A**) Additive gene transfer. Cells are transfected with a vector containing the gene of interest (GOI) and a resistance gene. After antibiotic selection the single cell clones are mixed and transferred to an enucleated oocyte (SCNT) followed by an embryo transfer (ET) to synchronized gilts. After genotyping and gene expressing analysis of the born pigs the best expressing animal is re-cloned or used for a second round of transfection. (**B**) Gene targeting. In the first targeting round the cells are transfected with a targeting vector containing beside homologous regions of the target locus, a resistance gene (RG1). After selection and characterization of the generated single cell clones, heterozygous knockout cells are used for SCNT followed by the ET. For the targeting of the second allele cells isolated of the heterozygous knockout animals are used for a second round of transfection, using a second targeting vector with another resistance gene (RG2). All following procedures resemble the first round.

For the generation of transgenic animals by additive gene transfer (Figure 5A), resulting in random integration of a gene construct into the genome, wild-type cells were transfected with a gene construct including the gene of interest and a resistance gene for the generation of stable transfected cell clones after antibiotic selection. Since a mixed population of transfected and selected cell clones was used for SCNT, the generated animals had to be examined for transgenesis and expression level in the organs and tissues of interest. The best expressing animals were used for breeding, for re-cloning or for a second transfection with further gene constructs to generate multi-transgenic animals.

In summary 7 different primary porcine kidney cell populations, including PKCm and PKC2109, isolated from neonatal to 13-month-old wild-type or transgenic pigs served as donor cells for SCNT after one or two nucleofection rounds with 11 different gene constructs [10]. In re-cloning experiments, 5 different PKC lines isolated from neonatal to 13-month-old cloned transgenic pigs without any further genetic modification served as donor cells for SCNT [10]. Overall the litter rate and size were satisfying, where 38 embryo transfers after cloning of transfected cells resulted in 22 litters (58%) with 83 animals and 35 embryo transfers after re-cloning gave rise to 16 litters (46%) with 48 piglets. Hence, different primary kidney cells, including PKCm and PKC2109, were capable of producing genetically modified pigs by additive gene transfer. The efficiency in transgenic pig delivery did not show significant differences between PKC2109, PKCm and the fibroblasts examined (M. Kurome *et al*., unpublished data).

#### Gene targeting

In contrast to additive gene transfer, where pools of stable transfected cell clones can be used for SCNT, gene targeting requires clonal selection, which means the generation of individual cell clones and their analysis for homologous recombination. Figure 5B shows the sequential gene targeting of 2 alleles for the production of a homozygous knockout pig. The PKCm line was used for targeting of 3 different loci [8], using linearized BAC-DNA. After a first round of transfection and selection, correctly targeted cell clones (1 allele) were used for SCNT. In summary, 19 ETs resulted in 7 litters (37%) with a total of 28 animals (including 1 stillborn) and 2 interrupted pregnancies (day 58 and 61) containing 17 fetuses. PKCs from heterozygous animals were used for targeting of the second allele of the locus. After transfer of embryos to 4 recipients 2 litters (50%) with 11 offspring (including 2 stillborn) were obtained. Thus, SCNT using targeted PKCm clones resulted in acceptable pregnancy rates and litter sizes.

## Discussion

Various types of primary transgenic or non-transgenic porcine cells were used as nuclear donors for the production of cloned embryos or piglets, but the characterization of the cells is normally restricted to the efficiency of transgenic animal production. Nonetheless, a comprehensive analysis of defined cell lines might facilitate choosing appropriate cell types for advanced transgenic strategies and improve the use of primary cells for the production of large animal models. We used two different primary kidney cell lines and compared them with fetal or ear fibroblasts and observed that subclones of these cells differ in morphology and growth potential. Various groups verified different types of fibroblasts (cortical and inner medullary fibroblasts) and other cell types such as dendritic cells, macrophages and lymphocyte-like cells in kidney cell cultures (reviewed in [26,27]). In our hands, the diversity of cell morphologies decreased over several passages in all investigated cell lines, probably due to the culture conditions promoting proliferation of few fibroblast-like cell types and a different lifespan of cultured cell types. Regarding the growth potential, PKCs grew faster than fetal or ear fibroblasts, although the kidney cells were isolated from 3-month-old animals, whereas PFF originated from a fetus and the PEF from a neonatal ear. The diversity of cell types in PKCs might influence the growth of cell population in a positive way due to factors which are produced by neighboring cells, e.g. the extracellular matrix produced by renal fibroblasts [26]. However, we showed that all investigated cells, including the kidney cells, grew better on coated compared to non-coated plates. It has already been shown that collagen type I, which is part of the extracellular matrix, is a prevalent substrate for the culture of several cell types, including human skin fibroblasts [28] and PFFs [7].

Various methods have successfully been used to introduce exogenous DNA into porcine fibroblasts. During chemical transfection, we suggest that the vesicles were formed of big DNA-lipid conglomerates and the vacuoles probably originate from cell stress during transfection. Overall, our lipofection results are very heterogeneous due to the numerous parameter combinations (ratio of DNA to lipid, amount of DNA, Plus-reagent and lipofection solution) which had to be tested. The tested chemical transfection methods and in addition the conventional electroporation were not suitable for efficient transfection either due to low transfection efficiencies, formation of vacuoles and lipids or high toxicity.

Nucleofection has been used for transfection of various cells [23,29]. We achieved best results by nucleofection of PKCs with 70% to 89% transfection efficiency, which is comparable to the efficiency of 90% in PFFs [30]. In general, after nucleofection of all primary cell lines (PKC, PFF and PEF) we observed low cytotoxicity and only marginal changes of cell morphology. This confirms partly previous studies using the nucleofection technology, showing no effects on cell properties, such as alteration of cell morphology, response to chemicals and pattern of gene expression [31,32].

Using PKCs as donor cells for SCNT, the obtained blastocyst rate of 21% was higher in comparison to other reports showing a blastocyst rate of 4 to 14.8% after 6–7 days [17,18] of embryo *in vitro* culture. The SCNT with PFFs as donor cells resulted in a blastocyst rate of 9.1%. In other studies the blastocyst rate using PFFs ranged from 11.9 to 31.2% [12,16,33,34], which is several times higher compared to our blastocyst rate. Nevertheless, it is difficult to compare cloning efficiencies between different laboratories, because a plethora of parameters influences the outcome of SCNT experiments. Even cell cultures from the same fetus may differ in their development capability of embryos after SCNT [35,36]. Moreover, the blastocyst rate is not compulsory meaningful for *in vivo* developmental competence, it rather gives a rough estimation of the principle applicability of a defined primary cell line as a donor for SCNT.

In the last years, the generation of cloned transgenic pigs using nucleofected fibroblasts from fetal or ear tissue was quite successful in our lab [9,10,37]. The usage PKCs for SCNT after transfection and for re-cloning [10] was in our hands satisfying and comparable to PFF, whereas it has to be kept in mind that different gene constructs were used. Furthermore, we were able to target successfully 3 different loci in PKCs by homologous recombination [8] and SCNT with several of these correctly targeted cell clones resulted in litter rates of 37 to 50%. Rogers *et al*. [7] verified that homologous recombination depends on donor cells, because they achieved targeting frequencies between 0.07 to 10.93% using various primary PFF cultures prepared from different fetuses, but from same uterus at the same time [7]. This underlines the importance of characterization of donor cell cultures provided for genetic modification and SCNT.

Beside the easy isolation procedure and good proliferation capacity of kidney cells it has to be mentioned that for the generation of multi-transgenic pigs and re-cloning kidney cells can be isolated after killing the animals for gene expression analysis anyway or from a biopsy taken from the living animal.

## Conclusion

Our porcine kidney cells are probably a mixture of different cell types showing better proliferation rate, growth capacity, transfection efficiency and blastocyst rate after SCNT compared to PFFs and PEFs. There is no evidence that this mixed population has a negative influence on transfection and further procedures which are necessary for transgenic pig production. Primary porcine kidney cells are highly suitable for additive gene transfer and gene targeting, and subsequent production of genetically modified pigs by SCNT. It has to be kept in mind that each primary cell culture originates from a different animal and preparation, and might show different properties. However, our dataset includes kidney cells from different animals and preparations all showing the same basic characteristics.

## Methods

### Animal care

All animal procedures in this study were performed according to the German Animal Welfare Act and to a protocol approved by the Regierung von Oberbayern.

### Cell culture

Porcine fetal fibroblasts (PFFs), porcine ear fibroblasts (PEFs) and porcine kidney cells (PKCs) were principally cultured as mentioned below unless not otherwise noted. Cells were grown on collagen type 1-coated (Serva Electrophoresis, Heidelberg, Germany) cell culture dishes, in medium containing Dulbecco’s modified Eagle medium (DMEM; Invitrogen, Darmstadt, Germany) supplemented with 293 mg/l L-glutamine, 100 units/ml penicillin, 100 μg/ml streptomycin (PAA, Pasching, Austria), 0.1 mM 2-mercaptoethanol (Sigma-Aldrich, Steinheim, Germany), 1% (v/v) nonessential amino acid and 1% (v/v) sodium pyruvate (Invitrogen) and different amounts of fetal calf serum (FCS; Invitrogen) depending on the primary cell line (PKC 10%, PFF and PEF 15% (v/v)). The cells were cultured at 37°C in a humid atmosphere of 5% CO_2_ in air. PFF and PEF were harvested using 0.1% trypsin (Difco^TM^, Becton Dickinson, Heidelberg, Germany)/0.008% EDTA (Sigma-Aldrich) solution and PKC were harvested with 0.4% trypsin/0.032% EDTA. The split ratio of the cells was between 1:2 and 1:4 depending on confluence and growth potential of the cells.

### Cell isolation

After slaughter the kidney (for PKCs) or the uterus containing the fetus (for PFFs) were excised surgically. The ear tissue (for PEFs) was obtained from a living animal by cutting a piece from the ear using a pincer. In general, tissue pieces were stored in washing buffer (PBS with 1-2% (v/v) Pen/Strep and 1-2% (v/v) Amphotericin B (PAA)) in the refrigerator or on ice until isolation. The isolation of the primary cell lines PKC2109 from a 3-month-old male pig and PEF0110 form a neonatal female piglet was conducted by the same way. Pieces taken from the cortex and medulla of the kidney (2×1×1 cm) and from the ear (0.5×0.5 cm) were washed twice in washing buffer, minced and washed with DMEM by centrifugation (5–10 min; 180×g) until the supernatant became clear. Subsequently, the pelleted tissue pieces were resuspended in 15 ml Hank’s Buffered Salt Solution (HBSS; PAA) with 0.1 % (w/v) collagenase II (Invitrogen) and incubated at 37°C while stirring for 1 to 1.5 h (kidney) or 2 h (ear). After incubation, flasks were filled up to 50 ml with DMEM, filtered through a 100-μm mesh and washed with DMEM (5–10 min, 180×g) until the supernatant became clear. Depending on pellet size 1/6 to 1/24 of the resuspended PKCs were seeded per 100 mm plate and all resuspended PEFs onto a 60 mm plate. The isolation of the primary cell line PKCm from the kidney (cortex and medulla) of a 3-month-old male pig was basically the same with the following modifications. The collagenase digestion was done at 37°C with shaking every 15 min, and after digestion the remaining tissue pieces were further treated using 0.25% trypsin/0.02% EDTA for 15 min at 37°C.

For the isolation of PFF26, the backbone of a 27-day-old male fetus was prepared by removing head, legs and internal organs and washed 3 times in PBS containing 1× Pen/Strep, minced and washed twice in DMEM. Tissue was resuspended and incubated in EGTA buffer [38] rotating in front of an infrared lamp at 37°C for 33 min. Afterwards, tissue pieces were centrifuged, resuspended in DMEM containing 1 mg/ml (m/v) collagenase II and 1 mg/ml hyaluronidase and incubated for 20 min at 37°C. After washing by centrifugation, tissue pieces were incubated in DMEM with 1 mg/ml (m/v) dispase and digested for 40 min at 37°C. Afterwards, the cells were washed by centrifugation, resuspended in DMEM, filtered through a tea strainer and washed twice in DMEM. The whole cell pellet was resuspended in culture medium and seeded onto a 100 mm petri dish.

### Chromosome preparation

The metaphase spreads were prepared according to standard protocols [39]. In brief, the cells were harvested with a confluence of 60-90%, incubated with Colcemide (Invitrogen) (10 μg/ml) for 1 h at 37°C, followed by hypotonic treatment (75 mM KCl) for 15 min at 37°C, fixed and washed 3 times with ice-cold fixative (75% methanol, 25% glacial acetic acid). Then, cell suspension was dropped onto 45°C preheated glass slide, dried and chromosomes were mounted with Vectashield antifade solution containing 4’,6’-diamidino-2-phenylindole (DAPI; Vector Laboratories, Burlingame, CA, USA) for counterstaining. Metaphases were analyzed using an inverted epifluorescence microscope (Axiovert 200M; Zeiss, Germany). Chromosomes were counted using the ImageJ software.

### Growth curve

5.5×10^4^ cells of the primary cell lines PKCm, PFF26, PEF0110 were plated onto 12-well plates and cultured under standard conditions. Over a period of 5 days, 3 wells of each culture were trypsinized and counted every 12 h. Population doubling was calculated by the following formula using the time between the 2 measurement points of 36 h and 60 h:

(1) $$\frac{\left[\text{log}\left(\text{counted cell number}\right)–\text{log}\left(\text{starting cell number}\right)\right]}{\text{log}\left(2\right)}$$

### Cell proliferation assay

Different cell numbers (2,000, 5,000, or 10,000) of PKC2109 and PKCm were seeded in duplicates onto 96-well plates 48 h before treatment with MTT. To obtain a standard curve with defined cell numbers, seven measuring points in duplicates were applied: 250–50000 cells per well (PKC2109) and 2500–50000 cells per well (PKCm). The cells were seeded onto the plate 4 h before the MTT treatment started, so that they had attached before treatment started. 10 μl of MTT (0.5 mg/ml) of the MTT-Cell Proliferation Kit I (Roche Diagnostics, Basel, Switzerland) were added to each well containing 100 μl culture medium and plates were incubated in the incubator for 4 h. After this, 100 μl of the solubilisation solution was added to each well and incubated in the incubator overnight to make the conversion of MTT visible. The formation of formazan crystals was measured by spectrophotometrical absorbance of the sample using Sunrise^TM^ microplate reader (Tecan Austria GmbH, Salzburg, Austria) at a wavelength of 562 nm. Analysis of data was processed with the Magellan Software.

### Cell transfection

For chemical transfections, 3.2×10^4^ of PKC2109 were seeded in 500 μl per well onto a 24-well plate the day before transfection. The cells were transfected with the GFP expressing plasmid pmaxGFP^TM^ (Lonza, Cologne, Germany) to determine the transfection efficiency. The medium was changed on the day after transfection. For evaluation of the transfection process different parameters were assessed 24 h after transfection (see legend of Tables 2, 3, 4, 5): the morphology and cell quality (rating: 1 to 5), the amount of cells in suspension, probably being dead cells (rating: 0 to 2) and the fluorescence intensity (score system: lowest [−] to highest [+++]).

### Nanofection

The Nanofectin Kit (PAA) was used according to manufacturer’s instructions. 0.5 or 1.0 μg DNA was added each to 1.2, 2, 3.2 or 4 μl Nanofection solution whereas 1.5 μg DNA was added to 2 or 3.2 μl Nanofectin solution.

### Lipofection

The Lipofectamin LTX + Plus Reagents Kit (Invitrogen) was used according to manufacturer’s instructions. 0.25, 0.5 or 0.75 μg of DNA was diluted in DMEM (5, 10 or 15 ng/μl DNA) and mixed thoroughly. The optimized volume of the Plus reagent (0.25, 0.5, 0.75 or 1 μl) was added to the diluted DNA. Lipofectamin LTX was added in different ratios (1:1 – 1:4) to the diluted DNA/Plus solution.

For physical transfection, cells were splitted 1 day before the experiment. Prior transfection cells were washed twice with PBS, trypsinized and counted. Then, 0.5 and 1×10^6^ cells were centrifuged (5 min, 180×g), resuspended in the respective solution and transfected either with pmaxGFP^TM^ or endotoxin-free purified pmaxGFP^TM^. After transfection, cells were seeded onto 35 mm or 60 mm petri dishes (depending on cell number) containing pre-warmed 15% FCS culture medium.

### Electroporation

For electroporation, cell pellets of PKC2109 were washed twice with PBS and resuspended in 600 μl of either Gene Pulser ^TM^ electroporation buffer (Bio-Rad, Munich, Germany), PBS or DMEM. DNA (1, 5, 10 or 20 μg) was added to the cell suspension and transferred into a 4 mm gap electroporation cuvette (Bio-Rad). Cells were electroporated with Gene Pulser II (Bio-Rad) using various settings (Voltage: 100 V, 230 V; High Capacity 500 μF).

### Nucleofection

For nucleofection of the cell lines PKCm, PKC2109, PEF0110 and PFF26 the Nucleofector^TM^II device (Lonza, Cologne, Germany) and the Amaxa^TM^ Basic Nucleofector^TM^ Kit Primary Fibroblasts (Lonza) were used. 0.5 or 1×10^6^ cells were mixed with 1, 2, 5, 10 or 20 μg of plasmid DNA and 100 μl Nucleofector solution and nucleofected according to manufacturer’s instructions with the recommended Nucleofector programs A24, T16, U12, U23 and V13.

If not stated otherwise, transfection efficiency was determined 24 h after transfection as the [(No. of GFP positive cells) / (No. of DAPI positive cells)] × 100. Therefore, 24 h after transfection, cells were washed twice with PBS and fixated with 4% (m/v) paraformaldehyde (Merck, Darmstadt, Germany) for 20 min at RT in the dark. After fixation, cells were washed with PBS and incubated with DAPI-Methanol (1 μg/ml) for 10 min at 37°C. Subsequently, cells were washed with methanol and PBS. Finally, the fixed cells were covered with PBS and analyzed using an inverted epifluorescence microscope (Axiovert 200M; Zeiss, Germany) equipped with an appropriate filter set.

### Additive gene transfer

Various cell lines of PKC, PFF and PEF were genetically modified by nucleofection using the Amaxa^TM^ Basic Nucleofector^TM^ Kit Primary Fibroblasts and the Nucleofector II® device (Lonza). 0.5×10^6^ to 1×10^6^ cells were transfected with 1–3 μg endotoxin-free purified and linearized conventional plasmids of 1.6 to 11.1 kb length and BAC vectors of >150 kb length. After transfection, cells were plated either onto 35 mm or 60 mm petri dish depending on cell number using 15% FCS in DMEM culture medium. After 24–48 h, selection was started with the following antibiotic concentrations: PKC – 10 μg/μl blasticidin S (PAA), 1.2 mg/ml G418 (Invitrogen) or 3 μg/ml puromycin (PAA); PEF – 4 μg/μl blasticidin S; PFF – 0.4 or 0.6 mg/ml G418. Selection was conducted for 7–10 days, including regular change of medium and 1 passaging step [10].

### Gene targeting

Endotoxin-free purified BAC vectors of >150 kb size were linearized and nucleofected into 0.5×10^6^ or 1×10^6^ PKC. 24–72 h after nucleofection the cells were counted and seeded onto 96-well plates for selection with 1.2 mg/ml G418 or 6 μg/ml blasticidin S in 15% DMEM culture medium for 7 days. Then, 96-well plates were screened for single cell colonies, which were splitted for DNA isolation and SCNT [10].

### Somatic cell nuclear transfer and embryo transfer

In general, 48 h prior to SCNT cells were cultured in starvation medium containing all components of the normal culture medium, but only 0.5% FCS. Nuclear transfer was performed as described by Kurome *et al*. [40] and Klymiuk *et al*. [10]. The generated embryos were transferred into estrus synchronized gilts (ET), as published before [41], on the same day of nuclear transfer or 1–2 days later. Gilts were checked regularly after ET by ultrasonic examination for conception and monitoring of pregnancy.

For determination of the competence of cloned embryos *in vitro* PKCm, PFF26 and PEF0110 were used as donor cells at passage 4 and transferred to enucleated oocytes. After electric fusion and activation of the resulting embryos, they were cultured in porcine zygote medium (PZM5) [42] for 7 days. Then, the quality of the embryos was evaluated, they were fixed with acetic acid and methanol (1:3), nuclei of embryos were stained with 1% orcein (Sigma) and counted thereafter.

### Statistical analysis

χ^2^-test was used to compare the rate of embryo development. The mean cell number of the embryos was compared using Student’s *t*-test.

## Competing interests

The authors declare that they have no competing interests.

## Authors' contributions

AR and AW conceived and carried out morphology, growth potential, transfection, additive gene transfer and gene targeting experiments and drafted the manuscript. MK, BK, VZ and HN were involved in nuclear transfer experiments. MK did the *in vitro* studies after SCNT and participated in embryo transfer. BK carried out embryo transfer and was responsible for animal care. NK conceived, coordinated and carried out molecular generation of constructs (plasmids and BACs) for additive gene transfer and gene targeting experiments and drafted the manuscript. EW coordinated and conceived pig projects, analyzed the data and drafted the manuscript. All authors read and approved the final manuscript.

## Supplementary Material

Additional file 1 **Single cell clone colonies of PKC2109 at P3. Single cell colonies were generated and analyzed after 5–8 days.** The cells and formed colonies differed morphologically: fibroblast-like cells [A, B, C, D, F], epithelial- and endothelial-like cells [E], cell size (smaller [A]), colony compactness (cells very close [A, C, E, F], gaps between cells [B, D]) and colony shape (clearly defined [A, C], frayed colonies [B, D, F]). Scale bar = 100 μm. Click here for file
