# Supplementary figures and images for: Potential of primary kidney cells for somatic cell nuclear transfer mediated transgenesis in pig
Source: BMC Biotechnol. 2012 Nov 9;12:84. doi: 10.1186/1472-6750-12-84 (PMC3537537; doi:10.1186/1472-6750-12-84)

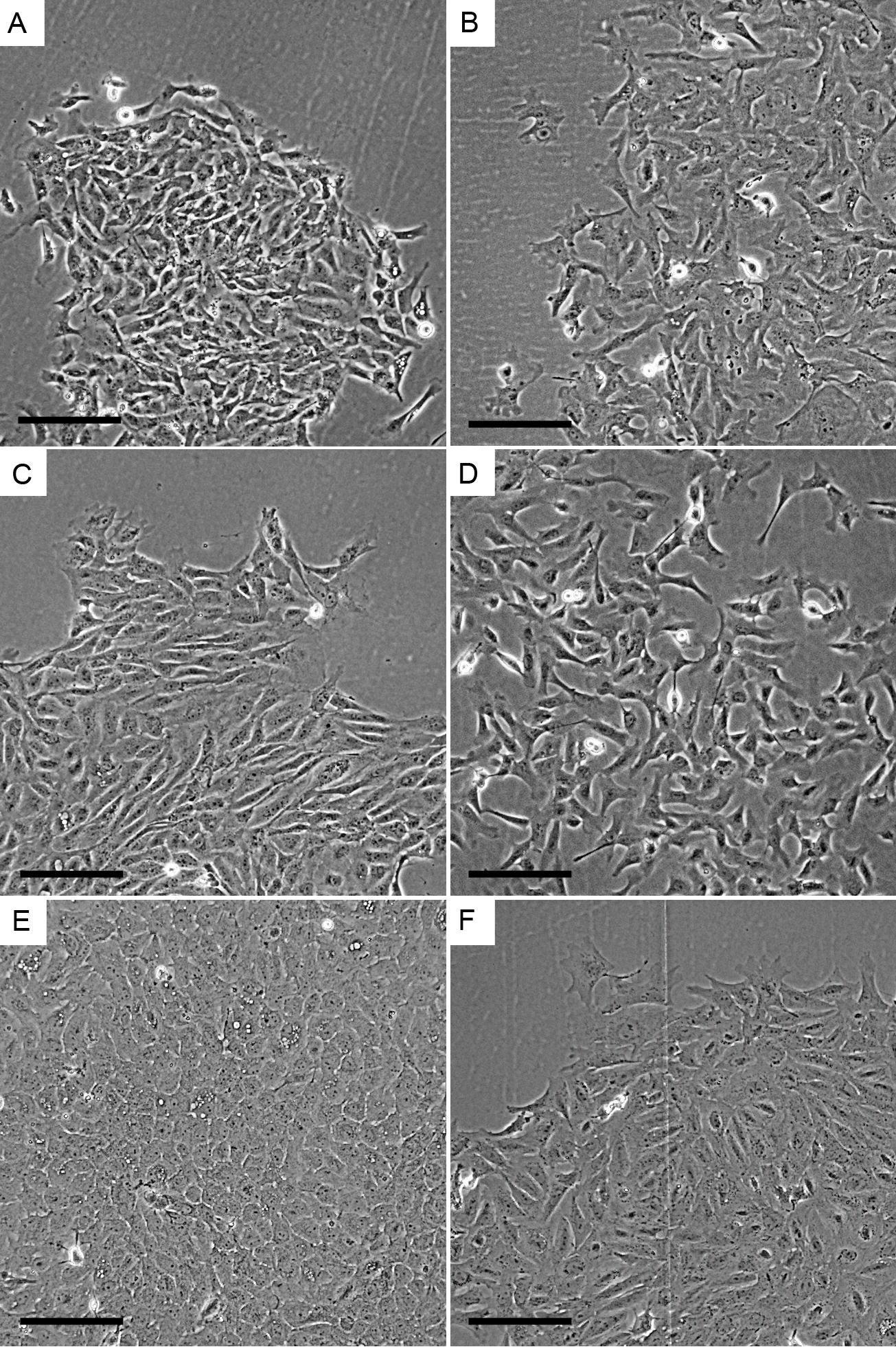

Supplement: Additional file 1 — Single cell clone colonies of PKC2109 at P3. Single cell colonies were generated and analyzed after 5–8 days. The cells and formed colonies differed morphologically: fibroblast-like cells [A, B, C, D, F], epithelial- and endothelial-like cells [E], cell size (smaller [A]), colony compactness (cells very close [A, C, E, F], gaps between cells [B, D]) and colony shape (clearly defined [A, C], frayed colonies [B, D, F]). Scale bar = 100 μm. [file 1472-6750-12-84-S1.tiff]
